# Can pragmatic research, real-world data and digital technologies aid the development of psychedelic medicine?

**DOI:** 10.1177/02698811211008567

**Published:** 2021-04-22

**Authors:** Robin L Carhart-Harris, Anne C Wagner, Manish Agrawal, Hannes Kettner, Jerold F Rosenbaum, Adam Gazzaley, David J Nutt, David Erritzoe

**Affiliations:** 1 Centre for Psychedelic Research, Imperial College London, London, UK; 2 Remedy, Toronto, Canada; 3 Department of Psychology, Ryerson University, Toronto, Canada; 4 Maryland Oncology and Hematology, Rockville, USA; 5 Aquilino Cancer Center, Rockville, USA; 6 Massachusetts General Hospital, Harvard Medical School, Boston, USA; 7 Neuroscape, Department of Neurology, Physiology and Psychiatry, University of California San Francisco, San Francisco, USA

**Keywords:** Real-world data, pragmatic trials, psychedelics, serotonin

## Abstract

Favourable regulatory assessments, liberal policy changes, new research centres and substantial commercial investment signal that psychedelic therapy is making a major comeback. Positive findings from modern trials are catalysing developments, but it is questionable whether current confirmatory trials are sufficient for advancing our understanding of safety and best practice. Here we suggest supplementing traditional confirmatory trials with pragmatic trials, real-world data initiatives and digital health solutions to better support the discovery of optimal and personalised treatment protocols and parameters. These recommendations are intended to help support the development of safe, effective and cost-efficient psychedelic therapy, which, given its history, is vulnerable to excesses of hype and regulation.

Close to one billion people are affected by mental illness and substance misuse worldwide. In many developed countries, mental illness ranks top for burden of disease (James et al., 2018), is more common, impactful and costly than other health conditions, and is a core component of overall health. The total cost of mental illness in the USA is estimated to be $2.5 trillion (Trautmann et al., 2016), the global antidepressant market is worth over $13.5 billion (Brandessence Market Research Company Pvt Ltd, 2019) and the wellness sector is estimated to be worth over $4.5 trillion (Global Wellness Institute, 2019).

Despite record increases in psychiatric medication prescription rates, the prevalence of mental illness is not reducing and may well be increasing in certain populations, such as the young (Twenge et al., 2019). There are indications that rates of mental illness have increased during the coronavirus disease 2019 (COVID-19) pandemic (Armitage, 2021; Inkster et al., 2020; Mahase, 2021). Evidence indicates that the efficacy of leading drug (Cipriani et al., 2018; Lewis et al., 2019) and psychological interventions (Flint et al., 2015) is modest, and there is scope for improved tolerability (Massabki and Abi-Jaoude, 2021) and access (Clark, 2011). Most mental health interventions are reactive. Effective prophylactic intervention would be hugely valuable (Patton et al., 2016). Relatedly, early life trauma (Varese et al., 2012) and mental illness (Copeland et al., 2009) are reliable predictors of future morbidity.

There is a legacy of division between the biological and psychological arms of mental health care and research. A notable initiative towards innovation in biomedical psychiatry is the Research Domain Criteria (RDoC) (Insel and Cuthbert, 2015). The main principle of RDoC is that, since diagnostic criteria are a product of clinical expediency, transdiagnostically relevant pathological mechanisms and treatment targets may have been overlooked. Relatedly, there is now good evidence for genetic overlap between psychiatric disorders (Brainstorm et al., 2018). RDoC is primarily a biological initiative that aims to translate mental illness into ‘brain illness’, for the purpose of discovering candidate brain biomarkers and treatment targets (Insel and Cuthbert, 2015).

Notable initiatives towards innovation in psychological health care include efforts to improve the cost-effectiveness of (Richards et al., 2018), access to (Moller et al., 2019) and reach of psychotherapy – e.g. through utilising technological advances (Leff et al., 2014; Richards et al., 2018) and social and familial networks (Freeman et al., 2019). So-called ‘third wave’ psychotherapeutic approaches have gained traction, e.g. with a spike in the popularity of mindfulness (Segal and Teasdale, 2018) and growing interest in – and evidence for – acceptance and commitment therapy (Hayes, 2019). Bearing in mind relevance to RDoC, one important characteristic of these approaches is their alleged transdiagnostic relevance, i.e. that they seek to identify and target a common pathological mechanism, but more work is needed to link the relevant psychological constructs, such as ‘psychological flexibility’, with biological processes.

There are promising signs of confluence between psychiatry’s biological and psychological divisions however, including a growing appreciation of the value of both psychological and neurobiological accounts of mental illness and its aetiology, as well as how environment, mind, brain and body interface and interact (Carhart-Harris, 2018) – consistent with the ‘biopsychosocial’ model (Engel, 1977; Wade and Halligan, 2017). Specific examples of biopsychosocial research in psychiatry include studying: (1) gene × environment (Caspi et al., 2010) and (2) drug × environment interactions (Alboni et al., 2017) – of which drug-assisted psychotherapy can be considered an example (Carhart-Harris, 2018), (3) neurophenomenology (Varela, 1996) and (4) the biological mechanisms of psychological interventions (Brooks and Stein, 2015).

Into this arena comes psychedelic therapy, a quintessentially biopsychosocial intervention. Evidence indicates that psychedelic therapy is a particularly promising and progressive mental health care solution (Nutt et al., 2020). Classic serotonergic psychedelics can be most precisely defined by their pharmacology, i.e. agonist action at the serotonin 2A receptor, which, if blocked, effectively abolishes their signature psychological effects (Nichols, 2017). Psychedelic therapy is defined here as psychologically supported classic psychedelic drug experiences – although we recognise that psychotherapy alongside experiences induced by certain other psychoactive substances, e.g. MDMA and ketamine, bears relation to classic psychedelic therapy.

Psychedelic therapy has shown promise for a range of different mental health conditions, including: (1) depression (Carhart-Harris et al., 2016; Davis et al., 2020), (2) end-of-life anxiety (Griffiths et al., 2016), (3) addiction (Johnson et al., 2014) and (4) obsessive compulsive disorder (Moreno et al., 2006). Indirect evidence also supports its potential for treating eating disorders (Renelli et al., 2020; Spriggs et al., 2020) and chronic pain (Whelan and Johnson, 2018). See Andersen et al. (2020) for a review.

The Food and Drug Administration has granted ‘breakthrough therapy’ status to two independent multi-site double-blind randomised controlled trials (DB-RCTs), aiming to bring psilocybin therapy to marketing authorisation for depression, while related work is currently underway across Europe. Population (Hendricks et al., 2015) and controlled studies (Griffiths et al., 2018), as well as large retrospective (Forstmann et al., 2020) and prospective surveys (Haijen et al., 2018), are generating evidence for improved mental well-being across a large demographic, potentially opening psychedelic therapy up to a sizeable wellness market. The successful initiative to legalise psilocybin therapy in Oregon, USA, intentionally included access for healthy individuals.

In addition to its putative transdiagnostic utility, other reasons to feel optimistic about psychedelic therapy include: its novel action (Carhart-Harris and Friston, 2019), and rapid and enduring therapeutic impact (Nutt and Carhart-Harris, 2021; Nutt et al., 2020). Unlike traditional psychiatric drugs, positive effects have been observed for several months after just one or two doses. In terms of safety, psychedelics such as psilocybin (‘magic mushrooms’) have a favourable toxicity profile and therapeutic index, and negligible addiction potential (Rucker et al., 2018). Not wishing to neglect rare cases of putative iatrogenesis, including those of so-called ‘hallucinogen persisting perceptual disorder’ (Rucker et al., 2018), the main hazards of psilocybin therapy relate to the intensity of the psychological state produced by higher doses, and associated need for a carefully engineered contextual container, e.g. with effective psychological preparation, supervision and aftercare.

The utilisation of a drug-induced period of heightened cortical plasticity is likely to be a core component of psychedelic therapy’s mechanism of action (Carhart-Harris and Friston, 2019) and candidate functional (Carhart-Harris, 2018a) and anatomical biomarkers (Ly et al., 2018; Xing et al., 2020) of this are already being examined. In the context of a predictive processing framework, the ability of psychedelic therapy to (1) relax and (2) (with psychological support) recalibrate cognitive and behavioural biases may be a central part of its action (Carhart-Harris and Friston, 2019) – as may an accelerated learning rate (Harvey, 2003).

How can we best advance the science of psychedelic medicine? Here we advocate pragmatic considerations (Purgato et al., 2015), the utilisation of ‘basket’ protocols (Parmar et al., 2017), as well as digitally aided data registries. Distinguishing pragmatic from confirmatory trials, the former refers to the actual, realistic (‘real-world’) conditions under which a therapeutic intervention will be received (e.g. when approved and marketed), whereas confirmatory trials typically engineer experimental conditions to support strong scientific inferences, but these often poorly reflect real-world conditions. Basket protocols are defined as single protocols, approved by relevant research regulatory bodies, that allow for a single intervention to be tested for multiple different (but often linked) disorders or conditions, such as a single drug for different types of cancer.

Developers may be right to adhere to convention in delivering confirmatory trials, but, if resources and conditions allow, considerable benefits could be gained from more explorative study designs. Such exploration may be best served by research that can address a greater range of questions of clinical and real-world relevance. Similarly, whereas confirmatory trials may choose to constrain eligibility and treatment criteria, pragmatic trials may benefit from broadening them, while maintaining a sensibly high-bar for (assumed) contraindication-related exclusion criteria. Easy to sample biometrics (e.g. via wearable devices or sensors and simple electroencephalography) and behavioural sampling (e.g. natural language and phone-use) could accrue large pools of objective data with potential predictive value.

If such studies and data registries are designed with careful consideration of data quality and fitness, pragmatic research could create significant value for various different stakeholders, e.g. scientists, clinicians, regulators, health care systems, payers and investors. As has been the case with medical cannabis, treatment-seeking patients will look for guidance from clinicians who take theirs from scientific evidence. Liberal policy changes occurring prior to the conducting of sufficient research could create similar problems for clinicians as occurred with medical cannabis. Such imperfect clinical scenarios could, however, also represent opportunities for innovative pragmatic and observational research. The creation of electronic data registries, e.g. for prescribers of psilocybin therapy in regions legally permitting (regulated) access (such as Oregon, USA), may be one appealing example, enabling the collection of valuable real-world data.

Data registries and pragmatic trials will collect data from broad and diverse samples. Data on use among healthy individuals can be supplemented by data from individuals seeking psychedelic therapy treatment for depression, particularly as safety is being established in this population (Carhart-Harris et al., 2016; Davis et al., 2020). An even more ambitious project would be to utilise a protocol to *only* exclude individuals where there are good reasons to suspect elevated risk and inadequate specialist support. Indeed, future psychedelic therapy clinics in areas supporting legal access and/or operating under a (pragmatic) research mandate may support such a scenario. Moreover, utilising digital tools, such as cellphone apps (e.g. mydelica.com), to track outcomes linked to psychedelic use could generate large data pools that could be mined to inform on such matters as patient screening and treatment optimisation.

Whether via data registries annexed to legal-access psychedelic therapy or approved pragmatic research trials, or both, the proposed approaches can serve the agenda of identifying transdiagnostic treatment targets (Carhart-Harris and Friston, 2019). The RDoC initiative pays selective attention to phenotypes associated with pathology, neglecting parameters associated with wellness, and this may be an oversight. Evidence of (1) reliable and sustained improvements in well-being (Haijen et al., 2018) and lifestyle (Ona et al., 2019) with psychedelic therapy, as well as the maintenance of psychological wellness (Ross et al., 2016), (2) recognition of the bidirectional relationship between psychological and physical health, and (3) awareness of the substantial costs required to implement any human drug study, let alone a clinical trial with a psychedelic, and (4) combined with a need for greater safety data across a diverse demographic, particularly given the liberalising political climate surrounding psychedelics, are all good reasons to justify innovative and pragmatic approaches to researching psychedelic medicine.

Collecting large sample sizes will enable better prediction-of-response modelling (Haijen et al., 2018), which will help mitigate risk and inform the potential customisation of care. A multi-site ‘trial’ or centralised registry would help generate and store the large data needed for reliable prediction-of-response modelling, with the added benefit of being able to assess between-site discrepancies and consistencies.

Confirmatory trials constrain important treatment parameters such as dosage and frequency of interventions, whereas pragmatic psychedelic trials could exercise flexibility here, particularly given the nascent nature of the treatment model, where practitioners cannot confidently claim to know the best parameters for all individuals and indications. In the context of psychedelic therapy, what dosage, frequency-of-dosing, as well as frequency and nature of post-dosing psychotherapy sessions are optimal, and for which cases, are all key questions that may be best addressed via pragmatic research under a basket protocol – and/or via digital data collection. Upper limits on the number of dosing sessions and lower limits on the intervals between them may be set to reduce the risk of bad practice, but redosing in response to relapse and based on clinical judgement may be permitted, thereby reflecting the conditions of clinical practice post roll-out. Most modern trials of psychedelic therapy have employed just one or two fixed-dose treatment sessions for *all* participants within relatively small and homogeneous samples, not because of assumptions about best practice, but because of alignment with regulatory traditions and budget constraints.

This article argues that carefully designed pragmatic trials implemented under a basket protocol could offer a powerfully progressive model for advancing our understanding of the safety, effectiveness, mechanisms, impact, best-use and pitfalls of a promising but vulnerable new treatment model in psychiatry. Progressive policy changes would likely be needed to actualise the proposed approach – but these are already occurring. For such policy changes to occur, a vision of the societal value of improved mental health care, and how this can be safely and effectively achieved via psychedelic therapy, will need to be well communicated to the public and policy makers.

For the time being, DB-RCTs will continue to sway sceptical opinions and aid progress with regulators, who presently base pivotal licensing decisions on data derived from such trials. Our view, however, is that data derived from pragmatic trials may be able to teach us *more* (than DB-RCTs) about how best to deliver the treatment and how it could impact on the lives of a broad cross-section of people. To be clear, the argument here is for the *complementary* value of pragmatic trials, not for their superiority over DB-RCTs. At the same time, however, we do challenge, as others have previously, the hierarchical preeminence of DB-RCT-derived evidence (Tucker and Roth, 2006).

Exploration has special value early-on in a learning process; thus, it seems prudent in the context of psychedelic therapy that it be given consideration now, rather than further down the development path, when suboptimal parameters begin to undergo regulatory ‘lock-in’. Rectifying this matter now may help mitigate risks associated with a too hasty scale-up of access. To achieve this, however, buy-in from multiple stake holders will be needed, including the public, policy makers and those in between, e.g. scientists, clinicians and investors. The motivation for doing so is to protect the sustainability of psychedelic medicine.

There are signs that modern psychiatry is ripe for a radical ‘new’ treatment model, and psychedelic therapy offers a multi-level paradigm-challenge. Assumptions challenged by it include those pertaining to: theoretical frameworks in mental health, models of therapeutic action, selection of sufficiently sensitive and specific assessment scales, trial design and clinical practice, plus drug, economic and social policy. Here we propose that pragmatic trials, data registries and electronic data capture will aid advances in psychedelic medicine by catalysing our understanding of best practice, which includes, but is not limited to, identifying and mitigating risks.

Pragmatic trials, data registries and digital and biometric data collection can interface well with so-called ‘*n* = 1 trials’ (Lillie et al., 2011), where individuals and/or prescribing doctors assess, prospectively, the impact of introducing a time-limited intervention (e.g. the A-B-A design) in single-case studies. In contrast to large-scale observational cohort studies that allow for the modelling and prediction of response across wide demographics at the cost of experimental control (Haijen et al., 2018), single-subject designs assess the effectiveness of an intervention experimentally, thus representing a scientifically rigorous alternative to RCTs (Lobo et al., 2017). When participants serve as their own comparison (measured under different experimental conditions over time), confounding variables such as age, gender or socioeconomic status are automatically controlled for, thereby decreasing the number of participants required to determine the likelihood of a causal relationship between intervention and outcome, and ultimately, research costs (Charness and Fehr, 2015).

Single-case approaches also bear relevance to ‘citizen-science’ initiatives, in which individuals’ willingness to engage in the scientific process is harnessed. For example, individuals may be invited to increase the rigour of their ‘self-experimentation’ by, for example, completing assessments, wearing biometric sensor devices or even engaging in a self-blinding placebo-controlled protocol, as was done recently for psychedelic ‘microdosing’ (Szigeti et al., 2021).

As implied by some recent studies of ours (Haijen et al., 2018), there is appetite for citizen-science-type engagement among users of psychedelics. Specifically, we foresee value in the use of smartphone apps to collect data pertaining to psychedelic use in a convenient and efficient way (see https://www.mydelica.com). For example, the combination of single-case trial designs and remote digital assessments could enable the collection of scientifically rigorous efficacy data on self-medicative psychedelic use in small or difficult to access patient populations (Elliott, 2002), the potential utility of which is particularly salient considering the significant challenges that COVID-19 has posed on clinical and research psychiatry, including psychedelic trials (Kelly et al., 2020). Data from *n* = 1 experiments can be aggregated and analysed using Bayesian statistics (Zucker et al., 2010) and multi-level regression and post-stratification analyses (Spiegelhalter, 2019) to identify meaningful relationships within potentially rich datasets that could ultimately inform effective future care strategies. Idiographic high-frequency time-series data collected through such methods (e.g. daily smartphone-based assessments) could enable more ecologically valid and nuanced modelling of change than conventional study designs (Hofmann et al., 2020).

Zooming-out, the highlighted approach should not be interpreted as implying relaxed standards of screening or scientific rigour in psychedelic research. We are not, for example, advocating that researchers relax contraindication-based exclusion criteria intended to mitigate risks of adverse responses. Some might feel it premature to propose pragmatic trials for psychedelic therapy, as these are typically reserved for treatments that are already incorporated into clinical practice. However, we believe that it is right to begin such trials now, as policy changes are already afoot and could ‘get ahead of the data’, as occurred with cannabis, for example. There is presently insufficient data on which to recommend specific treatment parameters for specific populations and indications, as well as ‘no go’ criteria (at least for a standard treatment approach) at screening, and big data pools would likely change this. Indeed, large-scale datasets from naturalistic sampling could have considerable harm reduction potential, by helping identify those most and least suitable for psychedelic therapy.

Progressive policy changes on psychedelic medicine will likely have trickle down effects on research, innovation and investment in psychedelic medicine, particularly in the implicated geographical locations. Given the considerable cost-implications of a multi-site pragmatic research programme, health care payers (e.g. insurance companies) and/or industry buy-in would likely be required to fund it, and the relevant parties would need to be incentivised to do so. Digital data collection could lessen this burden, however, particularly if individual end-users (i.e. psychedelic users themselves) feel sufficiently incentivised to engage directly, e.g. via inputting data via a phone app (https://www.mydelica.com).

Mainstream, institutional-level funding has still not come into psychedelic science; philanthropy and now commercial investment have been its main drivers. Increasing demand for psychedelic therapy is poised to synergise with an upswell in initiatives to meet this, potentially jeopardising standards of safety and professionalism if corners are cut. In anticipation of and, to some extent, already witnessing the beginning of a ‘hype-cycle’, we believe that innovative, pragmatic and exploratory research can play a vital role, helping safeguard the development of a particularly promising, yet vulnerable, approach to mental health care.
